# Optically Stimulated Luminescence Silicone Foils for 2D Dose Mapping in Proton Radiotherapy

**DOI:** 10.3390/ma18091928

**Published:** 2025-04-24

**Authors:** Michał Sądel, Leszek Grzanka, Jan Swakoń, Damian Wróbel, Sebastian Kusyk, Lily Bossin, Paweł Bilski

**Affiliations:** 1 Institute of Nuclear Physics Polish Academy of Sciences, PL-31342 Krakow, Poland; 2 Department of Radiation Safety and Security, Paul Scherrer Institute, Forschungsstrasse 111, 5232 Villigen, Switzerland

**Keywords:** two-dimensional radiation dosimetry, optically stimulated luminescence, proton radiotherapy, MgB_4_O_7_:Ce,Li, Gafchromic^TM^ EBT3 films

## Abstract

A novel reusable silicon foil dosimeter based on the new emerging optically stimulated luminescence (OSL) material MgB_4_O_7_:Ce,Li (MBO) is developed and characterized for dosimetric verification of spatially resolved radiotherapy doses. Direct comparison of the spatial (two-2D towards three-3D) proton dose mapping can be achieved with an appropriately designed optical detection setup equipped with a light source (e.g., LEDs) that illuminates the dosimeter and a highly sensitive CCD camera that simultaneously acquires the 2D OSL light from the foil. The newly designed (2nd generation) optical setup allows the registration of high-resolution 2D proton doses (below 0.1 mm resolution) and reconstruction of the 2D proton dose distribution with an accuracy comparable to that of the Gafchromic^TM^ foils, the current standard of passive 2D dosimetry in radiotherapy. This article outlines the technology’s potential application with respect to the commercially available Gafchromic^TM^ EBT3 films in measurements of the clinically relevant, spatial proton dose mapping. The obtained comparison of the proton radial dose profiles (for EBT3 films vs MBO foils) agrees within 5%. The resulting image resolution (0.074 mm/px for MBO foil) corresponded well with the tested EBT3 films (0.085 mm/px), indicating excellent properties for future 3D proton dose verifications of modern radiotherapy techniques (e.g., proton radiotherapy).

## 1. Introduction

With recent developments in modern radiotherapeutic (RT) techniques, such as proton therapy, where the range of the particle track is critical, and particles stop in the well-defined treated volume of the patient’s body, the demand for patient-specific dosimetric verification systems for quality assurance (QA) is increasing and becoming more stringent [1,2]. Especially in proton treatments of small tumors (e.g., eyeball tumors), the precision in delivering treatment planning dose is at the range of sub-millimeter resolution. Since state-of-the-art RT delivers high-resolution spatially designed dose distributions, the treatment plans should be verified using 2D or 3D dosimetry techniques. However, at the present stage of research, the clinically available dose-verification tools do not measure high-resolution 3D dose distributions, limiting the development of future treatment procedures. The currently available spatially resolved systems are based on silicone/gel dosimeters consisting of a radiosensitive volume, which changes their optical properties (optical density) when subjected to radiation [3]. After irradiation, the dosimeters can be read out using optical computed tomography (OCT), and the 3D optical density distribution is obtained using a complicated inversion algorithm, which is a time-consuming process. An example of 3D dosimeters is based on polymerizing [4,5,6,7] or radiochromic gels [8,9,10,11,12,13,14,15]. However, their clinical use has so far been limited, mostly because of the lack of reusability of the gel material and the complicated read-out procedure, which is time-consuming and requires much expertise in the clinic. Therefore, the current state-of-the-art tool for QA measurements in RT is based on 2D arrays of ionization chambers [16] or diodes [17]. However, in the case of 2D passive dosimetry systems, which are still very popular for routine QA, the use of radiochromic films, whose color intensity changes proportionally to the radiation dose they receive, allows quantitative spatial dose mapping [18].

An alternative approach for a passive dosimetry system is based on optically stimulated luminescence (OSL). The two most commonly used (e.g., for monitoring personal doses) are OSL materials in the form of OSL chips (available chips that do not exceed 10 × 10 mm^2^) based on aluminum oxide (Al_2_O_3_:C) [18] and beryllium oxide (BeO) [19]. The fast developments in the domain of the OSL technology and materials suitable for practical application have been driven by their favorable properties: reusability, high sensitivity (doses down to a few µGy), and linear dose response (up to approx. 100 Gy for AL_2_O_3_:C) [20]. OSL is based on a well-known physical phenomenon occurring in insulators: when exposed to ionizing radiation, specific metastable electronic states in the band gap are populated. Subsequently, this trapped charge population can be read out by subjecting the OSL material to a stimulation light, which triggers the release of charges from their traps, allowing them to recombine and produce luminescence, providing a reproducible signal proportional to the absorbed dose.

This study investigates the feasibility of using OSL silicone foils for 2D dose mapping in proton radiotherapy. The objective is to develop a reusable, high-resolution dosimeter that provides precise spatial dose verification. This approach is both innovative and highly relevant, as current dosimetry techniques, such as Gafchromic^TM^ films, are costly and single-use. In contrast, the proposed OSL-based system presents a potentially more cost-effective and sustainable alternative. By introducing this novel dosimetric method, the research aims to address a key gap in radiation therapy, potentially improving the accuracy and reliability of proton dose distribution measurements.

The general idea of 2D OSL dosimetry is based on manufacturing foil-shaped dosimeters by mixing an OSL powder material and a transparent matrix, creating a flexible and optically active foil sheet. Next, the spatial dose distribution can be measured by illuminating a foil with a laser or LEDs and registering the emitted luminescence with a CCD camera or other photodetector. Such application of OSL for 2D dosimetry was proposed more than 20 years ago [21], but more progress has been achieved in the last few years. One of the very promising recently published works concerns the use of silicone films containing OSL-active nanoparticles of LiBaF_3_ and LiF:Cu [22], which was upgraded to measure 3D dose distribution (from voxels measuring 0.8 × 0.8 × 1.0 mm^3^) with a statistical dose precision of 5% at 100 Gy dose levels [23]. In a similar manner, in our previous works, we demonstrated advantageous properties of the foils based on other OSL materials: first LiF:Mg,Cu,P [24] and later LiMgPO_4_ (in short LMP) [25]. Using the LMP-based silicone foils, we showed (for the first time) that a direct reconstruction of a real 3D proton treatment plan prepared for an eyeball tumor can be successfully measured [25].

Despite the progress achieved and several favorable features of OSL dosimeters, there are still some issues when it comes to using them as a real 3D dosimetry tool for QA applications. Firstly, the resolution at the level of only slightly below 1 mm is not sufficient for some RT applications, e.g., for small-field dosimetry of proton eyeball radiotherapy treatments, where a resolution of 0.1 mm is needed. Secondly, all of the mentioned OSL materials suffer from the effect of the decrease of efficiency for densely ionizing radiation (so-called quenching effect). This results in underestimating the measured proton doses, as the proton path through the matter is characterized by the high ionization density (Bragg peak).

In this paper, we address both these crucial issues. Here, we introduce an advanced silicon foil dosimeters utilizing a newly emerging optically stimulated luminescence (OSL) material MgB_4_O_7_:Ce,Li (in short MBO) [26], which exhibits only a minimal quenching effect and possesses other favorable properties [27]. MBO has an effective atomic number (Z_eff_ ≈ 8.4), which is close to that of soft tissue (Z_eff_ ≈ 7.4), reducing energy dependence in photon dosimetry and improving dose measurement accuracy. In contrast to BeO, MBO can be processed into thin, uniform films, facilitating applications in high-resolution 2D dosimetry. The material is chemically stable and non-toxic, which simplifies handling and device fabrication compared to beryllium-based compounds [28]. It also exhibits low fading over time, which contributes to the stability of the stored luminescence signal. Furthermore, MBO does not show pronounced trap saturation under repeated irradiation, supporting its use in measurements involving accumulated or high radiation doses. The new emerging MBO OSL material in conjunction with the newly designed 2nd generation optical setup (for the first time within this study), allowed us to achieve a resolution of below 0.1 mm and reconstruct the clinically relevant proton dose distribution with an accuracy comparable to that of the EBT3 films, the current standard of passive 2D dosimetry.

## 2. Materials and Methods

### 2.1. Fabrication of the 2D OSL Dosimeter

**Figure 1:**
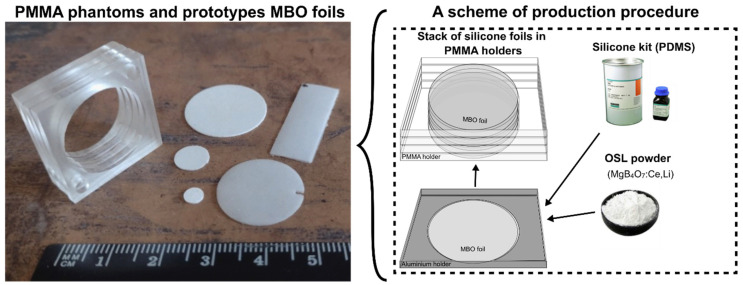
Sketch of the new production procedure consisting of the new prototype MBO flat sheet silicone foils of a size of 20.0 mm ± 0.1 mm in diameter and 0.43 ± 0.05 mm thickness, produced by homogeneously mixing the self-synthesized MBO [26] powder into the SYLGARD^®^ 184 silicone elastomer matrix at a 1:3 weight ratio and examples of the PMMA holders with a hole of the size of MBO foil, used for proton irradiations.

The prototype 2D flat sheet MBO silicone foils of a size of 20.0 mm ± 0.1 mm in diameter and 0.43 ± 0.05 mm in thickness have been developed according to the procedure described previously [25]. The changes introduced into the currently used dosimeter contain new OSL material with another size of OSL grain powder (sieved below 125 µm) mixed into a transparent silicone PDMS matrix at a 1:3 weight ratio. It should be noted that silicone is just a host for OSL material, allowing optical access to the grains, providing mechanical protection, and ensuring foil flexibility. It does not add additional optical signals during 2D read-out measurements. The used MBO material was doped with Ce (0.3 mol%) and Li (10 mol%) and synthesized at the Paul Scherrer Institute in Switzerland [26]. This material exhibits a fast luminescence emission, a wide linear dose-response range, and close to tissue equivalency, as highlighted in other studies [27,29,30,31,32]. Figure 1 shows the scheme of the production procedure and examples of prototype foils. The synthesis of MgB_4_O_7_ co-doped with Ce and Li was carried out using the solid-state synthesis method. During the material production, the following analytical grade reagents were used (all reagents from Alphatec Chemical Corporation, Quezon City, Philippines); Mg(NO_3_)_2_ (99.99% purity), H_3_BO_3_ (99.99% purity), Ce(NO_3_)_2_ (99.5% purity), and LiNO_3_ (99.5% purity). The solution synthesis involves mixing metal salts with distilled water, heating the mixture to 300 °C with stirring until a gel forms, removing the stirrer, further heating to 500 °C until the gel becomes dry and golden, and then grinding the dried material into a fine powder using a mortar and pestle. The detailed structural and morphological characterization of the material has been presented in the following studies [33,34].

### 2.2. The 2nd Generation Optical Detection System

**Figure 2:**
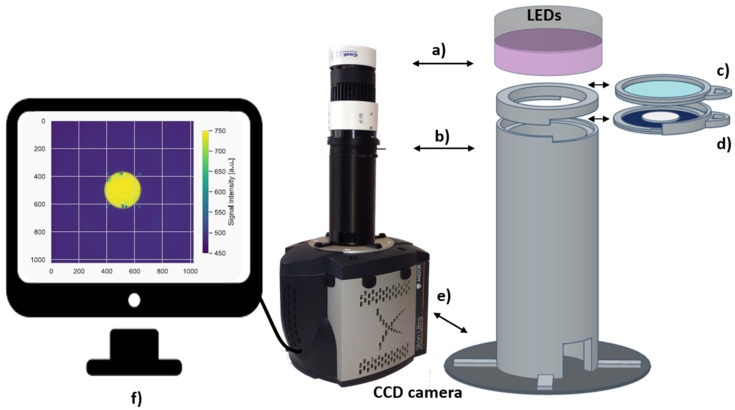
Sketch of the optical setup for retrieving the 2D OSL signal from the MBO foils, including (**a**) the CoolLED illumination system with the blue LEDs (440 nm with intensities 8 mW/cm^2^ at the sample’s position), (**b**) dedicated 3D printed optical set for appropriate light discrimination, including drawers (**c**) for LEDs light filtering (Chroma ET 440/40X filter and (**d**)) containing two filters; a Chroma Sloan-u BP filter and a Hoya U340 filter on which centrally placed MBO foils of a size 20 ± 0.1 mm in diameter and 0.44 ± 0.3 mm thickness, (**e**) the ANDOR iXon Ultra 888 back-illuminated EM-CCD camera with the attached Navitar 17 mm F/0.95 lens (hidden by the 3D printed tube), (**f**) an example image of the 2D OSL signal from the MBO foil irradiated with uniform source of Co^60^ gamma source after a dose of 20 Gy, captured by the camera-operated by µManager (version 1.53c) free software (for graph explanation, see Figure 4).

**Figure 3:**
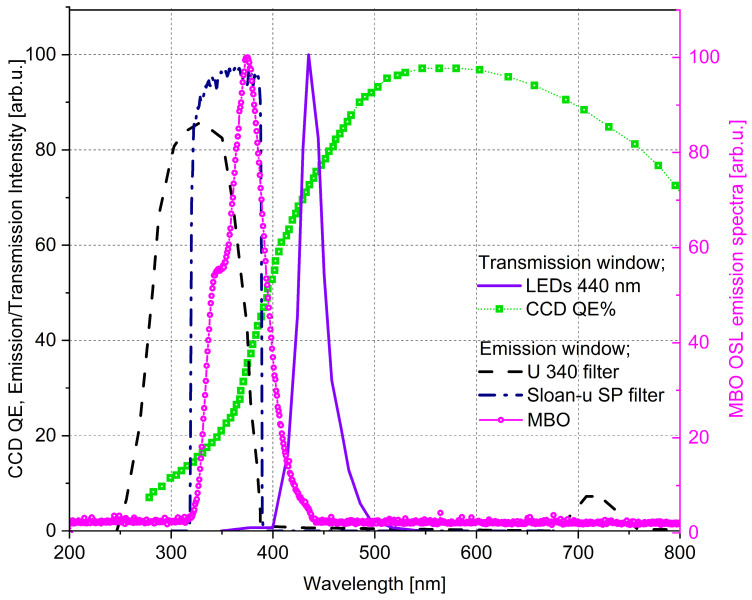
Comparison of the EM-CCD camera and PMT quantum efficiency, the LEDs emission spectra, the measured MBO foil emission spectrum, and the U-340 and Chroma Sloan-u BP filter transmissions (all left black scale). The measured MBO OSL emission spectrum (right magenta scale). The MBO emission spectrum extends from 320 nm to 440 nm, with a maximum peak at 374 nm.

The 2nd generation optical system is a newly designed system used for 2D OSL dose mapping in this study. It consists of the CoolLED pE100 light source (CoolLED, Andover, UK), which illuminates the foil area homogeneously with a 440 nm wavelength (with intensities 8 mW/cm^2^ at the sample’s position), a highly sensitive ANDOR iXon Ultra 888 back-illuminated EM-CCD camera (ANDOR Technology, Belfast, UK) supplied with a Navitar 17 mm F/0.95 lens (Navitar, Rochester, NY, USA), which images the MBO foil during light stimulation. Figure 2 shows the sketch of the optical detection setup for retrieving the 2D OSL signals. Easy handling operation and a fast, repeatable MBO foil read-out process are realized using a specially designed 3D printed tube connection (made with commonly used 3D filaments, e.g., polylactic acid—PLA filament) between the illuminating light source and the camera. The 3D printed tube includes easily changeable separate drawers, one for the filter set, which can be adjusted depending on the investigated materials’ spectral characteristics window, and the other with the place for the silicone foil size. The drawer’s shape ensures that the tested MBO foil sample is always best centered and aligned with the CCD sensor. The optical system parameters (imaging area of 1024 × 1024 pixels, with the camera matrix pixel size 13 μm × 13 μm), together with the applied lens for the camera and for the LED system, enable the read-out foil size of a maximum diameter up to 50 mm. Images were acquired using the µManager (version 1.53c), an open-source software [35] accompanying the CCD camera setup during an acquisition time of 30 s. The following filter set was chosen for light discrimination between excitation; band-pass filter Chroma 440/40 nm (Chroma Technology Corp., Rockingham, VT, USA) and emission light;a U340 Hoya filter (Hoya Corp., Tokyo, Japan) and an SP Sloan-u Chroma filter). The filter selection was chosen based on using the UV LED and the MBO OSL material emission spectra. The MBO spectrum was measured using the Ocean Optics QE Pro 00689 spectrometer (Ocean Optics, Dunedin, FL, USA) in a separate experiment with the blue LEDs (470 nm) and filter set to enable measurement below 450 nm. The measured spectrum extends from 320 to 440 nm, with a maximum peak at 374 nm (Figure 3).

**Figure 4:**
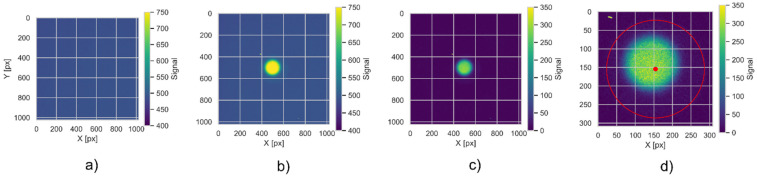
From left to right, the following processing steps are included: (**a**) background image, (**b**) after collimated (10 mm) 58.8 MeV monoenergetic proton beam for a dose of 20 Gy, (**c**) after background subtraction, (**d**) detector cutting and alignment (red line indicate the detector area).

### 2.3. Image Acquisition and Data Analysis

The 2D OSL images were acquired during the three-step procedure: 1st, during the background measurement, 2nd, during the ^60^Co gamma calibration campaign, and 3rd, during the experiments with the proton beam. Images are saved in the raw, lossless TIFF data format with a resolution of 1024 × 1024, single channel, and 16 bits per sample. Two images are acquired for each readout: live view (LV) with an image of the detector in visible light and raw signal (with OSL light emission from the detector). Further data analysis is performed using in-house developed calculation notebooks using the Python language (version: c15946). These are available in open repositories on GitHub (https://github.com/grzanka/osl_mb_foils, accessed on 17 April 2025). The workflow used in this work was adjusted to account for two factors as follows:

- circular shape of signal on the detector (due to collimator shape);
- 2D signal shape related to the readout system and light propagation in the tube;
- noise levels typical of the filters used.

**Figure 5:**
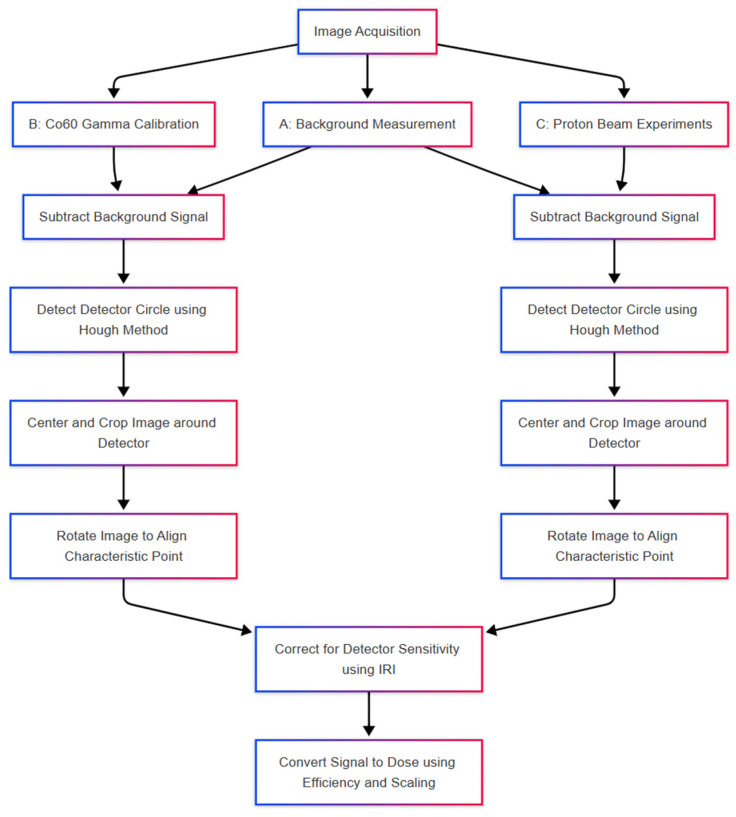
The block diagram of the image acquisition and data analysis procedure applied within the study.

The following steps were used in the data analysis within this study (also presented on the block diagram in Figure 5):

1. The raw TIFF files are being read into a 2D NumPy array (from an open-source numerical Python library) with signal encoded as unsigned 16-bit integers, using the imread function from the matplotlib Python library. These arrays are stored in convenient data structures (Python data classes), enumerated by a unique detector id.
2. The background signal is subtracted pixel-by-pixel from the raw data. All negative values are set to zero in the background-subtracted array. The background subtraction is performed separately for Co^60^ calibration data and for data from samples irradiated in proton beams.
3. The live view data is used to perform detector position based on the method described in [36]. We apply the Hough method of circle detection, which is parametrized to locate a single circle of radius corresponding to expected detector dimensions [37]. The boolean mask is automatically created, based on LV signal levels within 3 standard deviations of all the samples contained within a circle of 100-pixel radius, located inside the expected detector area.
4. In current experimental settings, the detector is visible as a circle with an area of 10% of the image. Therefore, after background subtraction, the images are centered on the detector’s central point and cropped so that the image frame is 20% larger than the detector radius. Further processing is done on the cropped image.
5. In the irradiation where the 10 mm collimator is used, we also locate uniformly irradiated areas on the detector. This allows for a spatial alignment as the gravity center of an irradiated part does not necessarily correspond to the detector’s geometrical center. This is performed using the centre of mass function from a script.ndimage Python module for image processing.
6. To account for various sensitivities in different areas of the foils, each of the foil samples was rotated. After rotation, the characteristic point, visible in the live image, was positioned in the top part of the image. Such an operation is necessary as the foils are being cut in a circular shape, making exact angular positioning difficult in the readout system. The rotation is performed automatically using custom code, which detects characteristic points and then rotates the image using the ‘rotate’ function from the Scipy image package [38].
7. The detector sensitivity correction is obtained from reference radiation in the form of Individual Response Images (IRI, as described in [25]). Finally, the signal images are divided by IRI images to correct for the detector sensitivity spatial distribution.
8. The last step of the data analysis is to translate the detector signal into the radiation dose. The detector signal is multiplied by the detector efficiency (as in [36]) and a scaling factor from the ^60^Co reference radiation.

The spatial distribution of the dose assessed with the MBO foils can be further used to assess detector signal linearity as the function of the dose and to study the spatial resolution of the obtained signal. In spatial resolution studies, we limit our considerations to the axially symmetrical beams. The resolution is characterized by measuring a radial dose profile (see Section 3.2).

### 2.4. Proton Beam Delivery and Reference Dosimetry

Proton irradiations have been realized at the Proton Eye Radiotherapy Facility at the Institute of Nuclear Physics, with a single proton beam of 58.8 MeV [39]. The proton beam dosimetry procedure was done in the same manner as described in [36]. The gamma reference dosimetry was carried out using the Marcus ionization chamber in a PMMA phantom constructed from 30 cm × 30 cm plates of different thicknesses. Irradiations were carried out using the ^60^Co gamma source from the Theratron 780E cobalt machine (Theratron, Ottawa, ON, Canada). The cross-check dosimetry was done using the Gafchromic^TM^ EBT3 films [40], with an in-house-developed calibration procedure based on red-channel readout and polynomial fit to the optical density response. The used EBT3 variant offers improved spatial resolution. During readouts, a resolution was set to 300 DPI, resulting in 0.085 mm/px scanner resolution, scanner model EPSON Perfection V850 Pro (Epson, Suwa, Japan). The EBT3 variant offers a dosimetric accuracy compared to previous generations, making it valuable for verifying and characterizing proton beam radiation delivery. The choice of EBT3 films was dictated by their wide adoption for routine 2D QA dosimetry in the radiotherapy scientific community [41].

### 2.5. Experimental Phantom Used for Proton Irradiation

**Figure 6:**
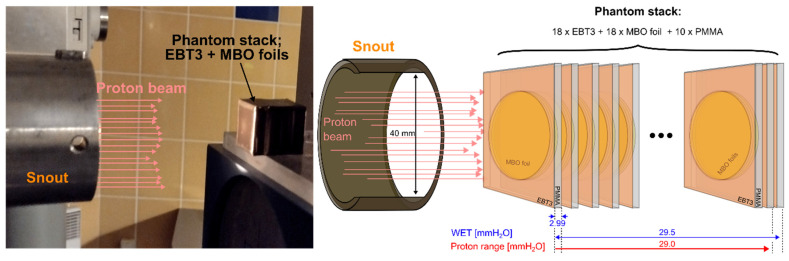
The experimental phantom used to measure the radial profile dose distribution for the EBT3 film and MBO foils. The setup comprised a combination of MBO and EBT3 films placed in a stack of a PMMA plate. The phantom protected with black tape was mounted in the isocenter of the therapy station on the Eye Therapy Chair in the proton treatment room of the Proton Eye Radiotherapy Facility (IFJ PAN) and irradiated with a monoenergetic 58.8 MeV single proton beam available from the AIC-144 isochronous cyclotron. The construction of the phantom comprised 18 EBT3 films, 18 MBO foils, and 10 PMMA plates. The total available range for protons of such a constructed phantom expressed in water millimeters fixed to 29.5 mm, covering the 58.8 MeV protons’ range, which is approx. 29 mm in the water range.

A specially designed phantom consisting of a stack of prototype MBO foils and EBT3 films was prepared (Figure 6). The phantom was constructed from PMMA square plates of 22 mm × 22 mm × 2 mm dimensions, with a hole tightly matched for MBO foils. In front of PMMA plates and MBO foils, the EBT3 films of the same size as the PMMA holder (22 mm × 22 mm × 0.3 mm) have been placed in selected positions. The phantom, consisting of 10 PMMA plates, 18 EBT3 films, and 18 MBO foils, folded tightly together and covered with black tape to protect against room light, was put on top of the therapeutic chair inside the treatment room during irradiations. The construction of the phantom is based on an experimentally determined water-equivalent thickness parameter (WET). The WET parameter relates to the thickness of the water layer, expressed in g/cm^2^, which causes the same loss of proton energy as in a given material with a given thickness. The WET parameters used in the phantom construction evaluated during separate experiments with proton beams are as follows: MBO foil (WET = 1.05), the PMMA plate (WET = 1.15), and the EBT3 film (WET = 1.31). The obtained values of the WET parameter and the number of used dosimeters (MBO and EBT3) resulted in an available range for proton propagation of such experimental phantom expressed in water millimeters to 29.5 mm, covering the 58.8 MeV protons’ range, which is approx. 29 mm in the water.

## 3. Results

### 3.1. Relative Luminescence Efficiency Response

Since the material luminescence efficiency depends on ionizing radiation density (i.e., kinetic energy, in case of irradiation with protons), the knowledge of MBO foil’s efficiency response to protons is crucial for any calibration procedure and for directly comparing the radial dose distribution profiles with that of EBT3 films. A detailed explanation of this parameter and the calculating procedure was provided in detail in our previous publication [36]. Here, we describe a similar procedure applied to the newly developed setup and MBO foils for an experiment with a collimated pristine BP with an initial energy of 58.8 MeV and an entrance proton dose of 7 Gy.

**Figure 7:**
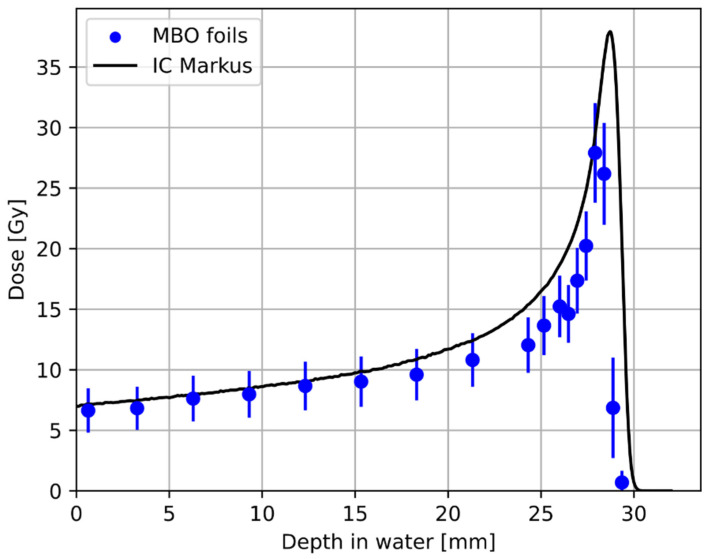
Comparison of the depth–dose response in water, measured for the Markus ionization chamber (solid black line) and the MBO foils placed in different depths in the unmodulated 58.8 MeV monoenergetic Bragg Peak. Data points denote mean values calculated on a circle with a radius of 90 px (~6.6 mm). The error bars on the MBO data points represent the spatial variability of pixel signals in the MBO foil. The dose was calculated by applying a ^60^Co calibration on a signal from proton irradiation. The distal part of the BP coincides with the last two experimental points from MBO foils.

**Figure 8:**
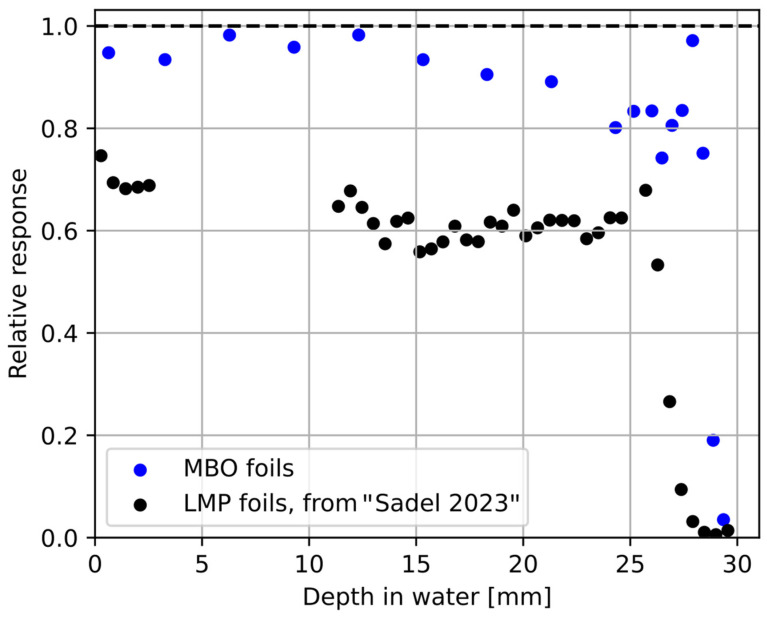
The MBO foils relative luminescence response (blue points) is calculated as the ratio of the dose obtained from the Markus ionization chamber (solid line in Figure 7) and the MBO foils (blue points in Figure 7). The last two blue points localized at the end of PB (for the 28 mm and 29 mm depth in water) represent the detectors where protons were partially stopped. The black points represent data for another previously tested LMP material according to [36]. For clarity, the error bars were not shown.

Figure 7 compares the proton depth dose distribution obtained with the Markus ionization chamber and the 18 MBO foils stacked together using the experimental phantom (Figure 6). The visible under-response of the MBO foils was calculated by applying ^60^Co calibration on a signal from proton irradiation, resulting in the so-called lower luminescent efficiency response (see Figure 8). The observed slight shift between the ionization chamber and the MBO foils may partially result from errors in the positioning either during the irradiation procedure or directly in the phantom stack. The error bars on the MBO data points represent the spatial variability of pixel signals in the MBO foils. Figure 8 shows the derived values of the MBO relative luminescence efficiency response to protons, which is the dose-response ratio obtained from the 18 MBO foils and the Markus ionization chamber, in comparison to the data calculated in the same manner for previously used LMP OSL material [36]. In general, the MBO poses only a minimal quenching effect compared to the previously tested LMP material. The efficiency response behavior is less pronounced at the proton entrance (higher proton kinetic energy), for which the response seems to be fixed around values of 0.95. In contrast, close to the end of the proton range (smaller proton kinetic energies), the efficiency decreases faster, reaching a value of 0.75 for the distal part of the BP. The mean relative efficiency of the MBO foils varies within ± 10% in the almost full available proton range (provided kinetic energy is large enough to fully penetrate the MBO foil thickness). For clarity purposes, the error bars were not presented in Figure 8. For a detailed explanation of the origin of error bars, please refer to the discussion section. 

### 3.2. Spatial (Axial) Resolution of MBO Foils vs. EBT3 Films

**Figure 9:**
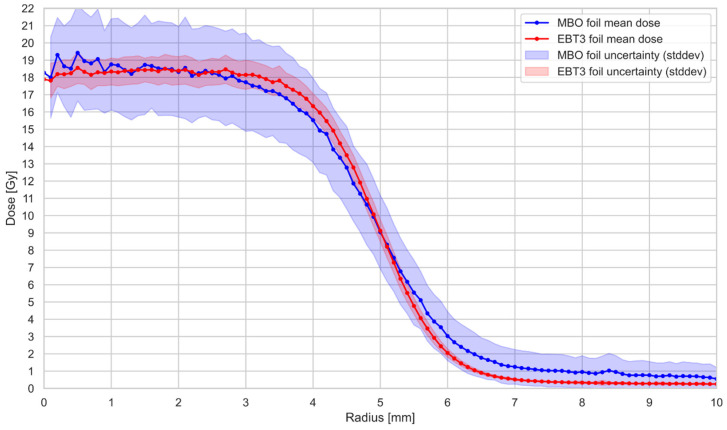
Comparison of the radial dose profile for EBT3 films (red line) and MBO foils (blue line) irradiated with 20 Gy of 58.8 MeV proton beam. Profiles were calculated by averaging pixel doses within concentric 0.1 mm rings centered on the gravity center of the dose distribution. Shaded bands represent measurement uncertainty (±1σ), calculated as the standard deviation of pixel values within each ring: red for EBT3 and blue for MBO. The center of the ring was taken as the gravity center of the dose distribution for each detector, respectively. Error bars presented here as the color bands were calculated as the standard deviation of doses from pixels within each concentric ring.

**Figure 10:**
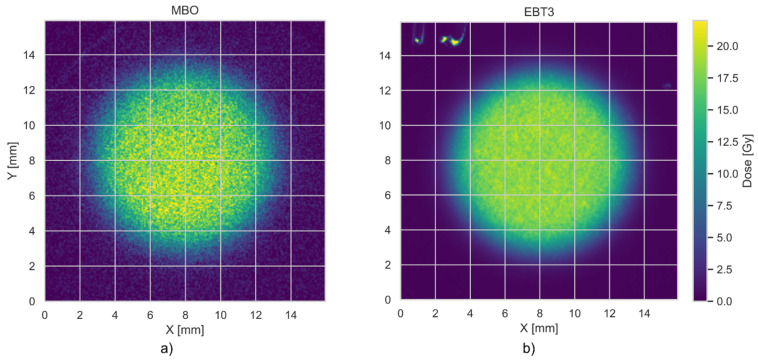
Comparison of the 2D dose response obtained for the MBO foil (**a**) and EBT3 film (**b**) irradiated together with a dose of 20 Gy with collimated (10 mm) 58.8 MeV pristine proton beam (see Section 2.4). The irradiation was performed at a water-equivalent depth of 2.6 mm.

**Figure 11:**
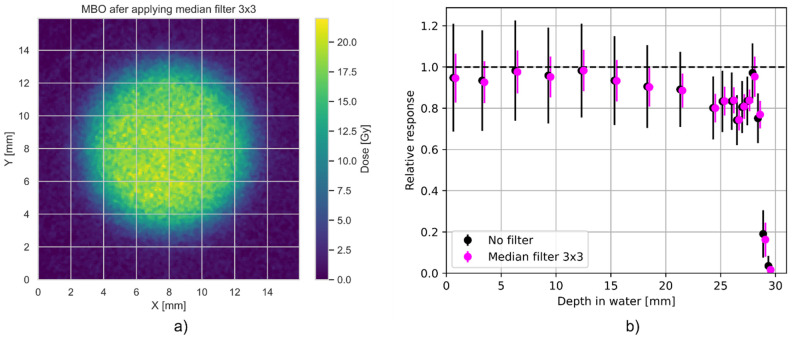
The dependency of MBO foil signal intensity after irradiation with a dose of 20 Gy of 58.8 MeV protons (see Figure 10a) after applying a median filter of a size 3 × 3 (**a**). (**b**) shows the relative response of the MBO foils (calculated as in Figure 8) with (magenta error bars) and without (black error bars) applying a median filter of size 3.

Figure 9 compares the radial dose profile for EBT3 and MBO foils, irradiated in one stack with a planned dose of 20 Gy of collimated (10 mm) 58.8 MeV proton beam. The irradiations were performed at a water-equivalent depth of 2.6 mm. Radial dose profiles were derived by averaging the signal intensity within concentric rings of 0.1 mm thickness, centered at the gravity center (centroid) of each detector’s dose distribution. This method assumes axial symmetry of the beam and uniform planar response of the detectors. The plotted profiles include shaded bands indicating the uncertainty of the dose values across each radius. These uncertainties are represented as ±1 standard deviation (1σ) of the pixel values within each concentric ring depicted in blue for MBO and red for EBT3. This reflects the degree of spatial variation in signal intensity within each radial segment rather than variability across repeated measurements. Figure 10 compares the 2D dose response obtained for the MBO foil (panel a) and EBT3 film (panel b). The MBO foil resolution calculated from the images acquired by the optical system setup (Figure 2) was 0.074 mm/px, corresponding well to the resolution of commercially used EBT3 films (0.085 mm/px, calculated for scanner resolution of 300 DPI). The obtained MBO spatial variability of the signal could be significantly reduced by applying median filters for each pixel, lowering the obtained error bars (see Figure 11). However, to keep the same image processing for both used MBO and EBT foils, we decided to perform all analyses without applying median filtering.

## 4. Discussion

To place the performance of the tested MBO-based OSL foils in a broader context, a comparative summary of different 2D dosimetry techniques is presented in Table 1. This table contrasts the optical and material characteristics of MBO foils, TL systems, and the widely used Gafchromic^TM^ EBT3 films. While EBT3 films offer high spatial resolution and are well-established in clinical settings, they suffer from dose saturation around 100 Gy and cannot be reused after exposure [34]. TL dosimeters such as LiF:Mg,Ti provide robust dose response but require thermal stimulation, which adds complexity to the readout process. In contrast, our MBO-based OSL system under development shows promising features, including high dose capacity (up to ~7 kGy [26]), minimal fading, reusability, and good tissue equivalency (Z_eff_ = 8.4) compared to the Al_2_O_3_:C (Z_eff_ = 11.3). The limitations identified in our optical readout setup (see Figure 8) highlight areas for further refinement, but the underlying material properties remain highly competitive.

The most challenging issue, as shown, e.g., in Figure 9, is the MBO foil uncertainty, which originates primarily from the capabilities of the optical detection system when measuring in lower dose regions, where the captured 2D OSL signal competes with the intrinsic background noise of the camera system. Pixel-to-pixel fluctuations for the currently applied optical setup may be significantly reduced by applying median filters. The error bars on the MBO data points are related to the spatial variability of pixel signals in the MBO foil dose, calculated by applying ^60^Co calibration on a signal from proton irradiation. The spatial variability of the signal can be significantly reduced by applying a median filter to the acquired image. However, this may come with some blurring of the image. To illustrate this behavior, Figure 11b shows the same data of the relative response of the MBO foils as presented in Figure 8, but with (magenta error bars) and without (black error bars) applying a median filter of size 3. After applying a median filter, the resulting spreads of pixel values and error bars were lowered by half. Consequently, Figure 11a shows an image of the same MBO foil irradiated with 20 Gy of 58.8 MeV protons, as presented in Figure 10a, after applying a median filter of size 3. 

The uncertainties associated to the readout could be reduced by improving the readout equipment (e.g., better isolation from external light sources, different optimized 3D-printed optical setups to increase light propagation together with different sequences of optical elements or by increasing the sensitivity of the embedded into to the silicone matrix the OSL material and the work in these directions is underway [26,34]. It should be noted that MBO is a new material that is still in the development phase, and a significant improvement can be expected by optimizing the preparation procedures [33].

It is important to note that the light distribution in the current readout system was optimized for a large foil size (approx. 50 mm in diameter). Therefore, for smaller foil sizes, the OSL signal at the foil edges is affected by light scattering during the optical readout in the system. One issue to mitigate it is to improve holder construction labels to match adequately for smaller (<25 mm diameter) foil readouts (see Figure 2d). This will be tested in the next step of the studies during the planned experiment with a patient collimator and a proton treatment plan prepared for a real eyeball tumor, similar to our previous study [25]. The expected 3D proton dose response comparison with the treatment plan is anticipated to show a similar response due to only a minimal quenching effect from the MBO foils, as shown in Figure 8.

Compared to the single example found in the literature [42], which utilized EBT3 films and 2D OSL foils for X-ray dose mapping, a good agreement was found in the high-dose areas. However, both detectors underestimated the dose calculated by the treatment planning system in the out-of-field regions. In this study, similar discrepancies were noted in the out-of-field areas, indicating that more careful calibration is needed for low doses (<7 Gy). Furthermore, when comparing the OSL foils with the EBT3 films, it’s important to remember that EBT3 is a single-use detector; once irradiated, the film cannot be reused [43]. In contrast, the newly developed MBO foils can be reused through a proper annealing procedure without losing their properties [27]. Additionally, using EBT3 foils in clinics is limited to 100 Gy, as the optical density saturates at higher doses. The MBO OSL detector, on the other hand, can register much higher doses (with a saturation limit of approximately 7 kGy [26]) while maintaining a similar spatial resolution to that of the EBT3 film.

## 5. Conclusions

We present a novel reusable silicon foil dosimeter system based on the new emerging OSL material MgB_4_O_7_:Ce,Li, which exhibits only a minimal quenching effect. In conjunction with the newly designed 2nd generation optical setup, they allowed us to achieve a resolution of below 0.1 mm and reconstruct the clinically relevant proton dose distribution with an accuracy comparable to that of the EBT3 films—the current industry standard of passive 2D dosimetry.

The characteristics of the technology were obtained by measuring the MBO foil’s luminescence efficiency to 58.8 MeV protons relative to ^60^Co gamma rays reference radiation. The obtained efficiency response agreed with the previously published data [27] and exhibited much less pronounced energy dependency than our previously tested materials [36]. The readout system capabilities were tested by measuring spatial resolution in axially symmetrical fields for MBO foils. The obtained comparison of the proton radial dose profiles (for EBT3 films vs MBO foils) leads to the conclusion that even for a small proton field (10 mm diameter), the dose penumbra agrees within 5%. The obtained image resolution (0.074 mm/px for MBO foil) corresponded well with the tested EBT3 films (0.085 mm/px), indicating excellent properties for future 3D proton dose verifications directly into the clinics.

In conclusion, it is essential to highlight that registering a delivered spatial 2D dose mapping in a quick and easy readout procedure (based on OSL phenomena) with high spatial resolution (below 0.1 mm) makes of the technology one of the leading tools for future 3D clinical dosimetry applications. What is also worth highlighting is that the 2nd generation of the optical setup tested in this study was constructed using readily available commercial components. These include the illumination LED system, the EMCCD camera, the filter set, and the open-source µManager software. Furthermore, the 3D-printed tube assembly (with the easily changeable optical drawers, holding filter set and OSL sample foil—see Figure 2) can be easily adjusted to accommodate various optically active dosimeters demonstrating the OSL phenomenon, enhancing the system’s functionality even more.

To summarize, the precise spatial characterization of MBO 2D foils presented in this study directly supports their potential application in radiotherapy dosimetry, where high-resolution dose mapping is critical for accurate treatment delivery. In advanced techniques such as IMRT and VMAT, even small spatial dose deviations can lead to significant under- or over-dosage in target or healthy tissues, affecting treatment efficacy and safety. By enabling sub-millimeter spatial resolution with reproducible luminescence response and near tissue equivalency (Z_eff_ = 8.4), the characterized material provide a technical basis for integrating material science advances into clinically relevant dosimetry tools [44].

## Figures and Tables

**Table 1:** Comparison of key characteristics of Optically Stimulated Luminescence (OSL), Thermoluminescence (TL), and Radiochromic (EBT3) film-based dosimeters. The table highlights aspects relevant for 2D spatial dosimetry, including materials used, operational principles, dose response range, stability, and limitations, focusing on magnesium borate for OSL applications.

Aspect	OSL	Radiochromic Films (e.g., EBT3)	TL
Principle	Luminescence induced by optical stimulation	Polymerization-induced color change	Luminescence induced by thermal stimulation
Materials	MgB_4_O_7_:Ce,Li	Poly-diacetylene or leuco dye-based films	LiF:Mg,Ti
Dose Response Range	Linear from 0.1–several kGy	Typically 0.01–8 Gy	Linear range 0.01–several Gy
Fading Characteristics	Minimal fading (<1% over 40 days)	Stable post-irradiation; some variation over long-term storage	Low fading (<3% over 1 year)
Advantages	High sensitivity, tissue-equivalent (Z_eff_ ≈ 8.2), fast signal decay	High spatial resolution, no readout stimulation needed	High sensitivity, robust materials
Limitations	Requires optical stimulation system; some trap instability	Sensitive to light/temperature; slower scanning process	Requires heating; signal can degrade if mishandled

## Data Availability

The computer code used in data analysis and interim data files are freely available in the public repository https://github.com/grzanka/osl_mb_foils (accessed on 23 January 2025). Raw data are available upon request.
